# A Digital Mobile Community App for Caregivers in Singapore: Predevelopment and Usability Study

**DOI:** 10.2196/25679

**Published:** 2021-05-26

**Authors:** May O Lwin, Anita Sheldenkar, Chitra Panchapakesan

**Affiliations:** 1 Nanyang Technological University Singapore Singapore

**Keywords:** caregiving, technological solution, mobile application, easy communication, caregiver, mobile app, communication, elderly, aging population, internet technology, community network, network

## Abstract

**Background:**

With increasing life expectancy and aging populations, the global prevalence of chronic diseases and the long-term care required for people with comorbidities is rising. This has led to an ever-growing need for caregiving. Previous literature has shown that caregivers face problems of isolation and loneliness. However, many health organizations mainly focus their efforts on in-person community groups that require participants to meet physically. This is not always convenient or accessible for caregivers who are often juggling caring for their care recipient with family and work responsibilities.

**Objective:**

With medical advancements such as the proliferation of mobile phones and internet technology, caregivers may have opportunities for easier access to resources and support. Technological innovations could help empower the caregiving community to seek assistance for improving their quality of life at their convenience. A community network app called Caregivers’ Circle was conceptualized in response to the needs of the caregivers on a day-to-day caregiving journey. This paper traces the predevelopment inquiry and technical details of this app to provide a clear understanding of its implementation along with a usability study to gauge user opinion of the app within Singapore.

**Methods:**

A predevelopment survey was conducted to identify specific needs of caregivers and gaps in the currently available web-based community networks. The survey consisted of questions on demographical data, health-related issues of the care recipient, mental and physical health–related issues of the caregiver, digital media use, information seeking, and support. This pre–app development survey was completed by 103 caregivers. Qualitative enquiries were also conducted with caregivers within Singapore to identify issues related to caregiving, support provided, and what caregivers would want from a caregiving mobile app.

**Results:**

From the feedback garnered from the caregivers, the developers were able to identify several caregivers’ needs and gaps within the current support networks. This feedback was integrated into the mobile app called Caregivers’ Circle upon development. The features of this app include a public *forum* for community discussions, a *marketplace* to buy and sell items, *care groups* to hold private discussions with friends or other users of the app, and a *friends* feature to search and add new caregiving friends.

**Conclusions:**

In general, the caregivers liked the Caregivers’ Circle app and were confident that this app could help them have a better quality of life. The Caregivers’ Circle app is unique in its integrated approach. The integration of many features that caregivers need on a daily basis into an easy app can save their time as well as help them navigate their life smoothly.

## Introduction

With the increase in life expectancy and aging populations, the global prevalence of chronic diseases and the long-term care required for people with comorbidities is expanding. This demographic shift has led to an ever-growing need for caregiving. A caregiver can be defined as “a person who provides direct care (eg, for children, older adults, the chronically ill)” [1]. In this study, a caregiver is defined as a person who provides direct care for individuals (eg, children, adults, older adults) with special needs or mental or physical disability. Although there are facilities such as care homes to provide for those who need assistance, there are many people who informally care for their loved ones. For example, it is estimated that the annual number of informal carer hours provided for people with dementia living at home globally was 82 billion hours in 2015 and this number is expected to increase [2]. Another type of caregivers are parents caring for children with special needs, and they are often the primary or sole caregivers. For instance, autism spectrum disorder was ranked among the 20 leading causes of years lost due to disability globally, with 3.6 million children aged 1-9 years living with this disorder. Autism spectrum disorder is only one of the numerous disabilities and conditions that afflict millions of children and requires long-term care [3].

Several studies have shown that caregivers face problems of isolation and loneliness. For example, Pertl et al [4] found that the high level of burden in dementia caregivers is associated with isolation and loneliness rather than the level of disability or the length of time that they have been taking care of the care recipient. A number of studies have shown that informal caregivers of children with intellectual disability, for example, would self-stigmatize themselves, which was reflected in the reactions to negative public response to the disability, by hiding their issues and withdrawing from social interactions [5,6]. Apart from adapting to the steep learning curve of taking care of patients, caregivers may have to adjust their own lifestyles, which include sacrificing their working hours, personal time, and relationships in order to help the patients. A research studying informal caregivers for the older adults within the United States found that over one-third of the respondents had quit or retired early from their employment to focus on caregiving. Among those who were still working, over half reported that caregiving had affected their work life [7]. Isolation and loneliness have been found to affect emotional and physical well-being and can lead to an increased likelihood of illness and death [8]. These factors, in turn, may also affect the care recipients and the quality of care they receive. Osborne et al [9] found that the poor mental health of caregivers negatively affected the development of their children diagnosed with intellectual disability.

With the current COVID-19 pandemic outbreak, these feelings of isolation have exponentially increased across the world due to the enforcement of social distancing and quarantine both in the general population and, particularly, in people with disabilities and their carers [10]. This has led to an increase in mental health issues and a need for coping strategies to deal with the impact of these social restrictions and the other potentially isolating measures in place to prevent the spread of the pandemic that currently remains prevalent with no foreseeable end.

Within Asia, cultural norms and filial piety often create expectations for family members to become the primary caregivers [11]. Southeast Asia is estimated to house 7.7% of the global total of people with dementia, 98% of whom are living at home, whereas only 55% of patients with dementia live at home in Western Europe. The cultural expectations that care needs be provided within families can lead to increased pressure and stress on carers who feel the need to become the sole carer, regardless of whether they feel capable, leading to an increased likelihood of ill health [2]. Lim et al found that in Singapore, caregivers of patients with cancer had lower quality of life scores than those in Western countries [12]. In 2010, 8.1% of Singaporeans aged 18-69 years stated that they were caregivers to friends or family [13], and this number is steadily increasing. Additionally, social stigma surrounding disability in Asia is much more prevalent. Many parents of children with disabilities or special needs attempt to hide the fact that their child may have special needs. Therefore, it is imperative that support is provided to enhance coping, improve quality of life, and reduce the burden of caregivers. Social support has also been found to reduce caregiver burden both in terms of family members and peers [14,15]. Several studies have reported that working with peers with similar problems enables the caregiver to feel less isolated and lonely. For example, parents of children with autism in China said their peers with similar experiences were less discriminatory and encouraged open discussion, which boosted spirits. The literature also suggests that providing support in the form of education and training can help to reduce this burden [16].

Both globally and within Singapore, several organizations and public health initiatives are focusing on improving caregiver well-being. In 2018, the Singapore Ministry of Health developed a Caregiver Support Action Plan [17] to provide financial support, training, and information for caregivers. Organizations such as the Agency for Integrated Care [18] and the National Council of Social Services [19] also offer assistance. However, many organizations mainly focus on in-person community groups that require participants to take time off to meet in the specified venue. This is not always convenient or accessible for caregivers, who are often juggling caring for their care recipients, in addition to family and work.

With the proliferation of mobile phones and digital technology, caregivers could have easier access to resources and support. Singapore has a mobile penetration rate of 86%, which is ranked the highest rate in Southeast Asia. Approximately 77% of the nation’s population are active social media users, which places Singapore among the top 3 countries globally for social media penetration [20]. Therefore, utilizing opportunities afforded by technology could assist in supporting the caregiving community. A review by Marasinghe et al [21] found that technology can reduce the burden of caregivers by assisting with the functional limitations of the care recipient. Technology-based support can also reduce caregiver burden by providing social support at low cost and accessibility to the caregiver [22]. For example, Damianakis et al [23] found that using a web-based community support group for caregivers of people with traumatic brain injury was positively received by participants. Piraino et al [24] found that personal web-based networks have the potential to improve family engagement and support by filling communication gaps that other traditional communication methods may not address.

Considering the issues faced by the caregivers and the increase in technology, we developed a community network mobile app for caregivers in Singapore with the aim of providing a space for caregivers to discuss problems, support each other, develop friendships with like-minded individuals, and ask questions to peers facing similar hardships. By creating a platform for caregivers in Singapore, we hope the app can meet the needs of the users through several features, including forums, a marketplace, private chat groups, and friend additions, to ultimately reduce the feelings of isolation and loneliness and provide locally relevant support. In this paper, we provide the technical details of the app development to provide a clear understanding of its implementation along with a usability study to gauge public opinion of the app.

## Methods and Results

### Pre–App Development Requirements Analysis

To identify the specific needs of caregivers and gaps in the currently available web-based community networks, a predevelopment survey was first distributed to caregivers in Singapore through web-based survey platforms, namely, Qualtrics and Google Forms or through face-to-face interviews. Participants were recruited through caregiving networks such as community centers, caregiving organizations, and special needs schools. Participants were eligible if they were between the ages of 21 years and 70 years. The survey questions were in English and Mandarin, which are the 2 official written languages of Singapore. Questions were based on demographics, health-related issues of the care recipient, mental and physical health–related issues of the caregiver, digital media use, information seeking, and support. Data were downloaded from the survey platforms into Microsoft Excel. For face-to-face interviews, the data were manually entered into Excel. All data were analyzed in SPSS (IBM Corp). A subset of the participants recruited for the quantitative survey also agreed to take part in face-to-face interviews to identify issues related to caregiving, the support currently provided, and what caregivers would require from a caregiving mobile app. Interviews were conducted in either English or Mandarin based on the preference of the participant. Participants consented to be audio-recorded and the interviews were then transcribed verbatim.

### Pre–App Development Surveys and Interviews

The pre–app development survey was completed by 103 participants. The demographical data are provided in Table 1. Approximately half (52/103) of the respondents were caring for recipients younger than 18 years, while 23.3% (24/103) were caring for recipients older than 70 years. The results showed that two-thirds of the caregivers felt stressed and lacked support in caring for their care recipient. About 38.8% (40/103) of the participants felt that their health has suffered since becoming a caregiver.

We conducted interviews with 29 participants (Table 1). The interview results supported the quantitative survey and found that many caregivers lack support and feel stressed about their situation.

…I think the caregivers' stress is probably something that er, that we all, a lot of us need help in, because most of us feel very alone in that situation.

Daughter of an 80-year-old patient with dementia

…You're always wondering whether you're doing enough. Um, or that, you know, I'm not doing a good job. Because there are good days and there are bad days, and then on bad days then you think that oh, you know, I am so bad at this.

Daughter of an 80-year-old patient with dementia

…sometimes it becomes very stressful, when I come home I become very tired, there are small things at home and you still have to handle your responsibility, it becomes very stressful. Talking to a therapist is a luxury, I think in Singapore it’s not that common, you go in there’s somebody that lets you say and then try to understand what you are feeling and guide your thinking and I think in Singapore my conception is this is very rare.

Father of a special needs 8-year-old child

…To me the mental health for caregiver is really important, sometimes I need to remind myself, I need to take care of myself in order to take care of my son, but this is hard.

Mother of a 10-year-old autistic child

Although several caregivers use web-based forums or communication channels to converse with peers in similar situations, many of the available forums are either global or consist of caregivers in Western countries, several of whom discuss matters related to social services and the available assistance within their countries. This makes it difficult for a Singaporean audience to completely relate to or utilize such recommendations.

…sometimes you see parents asking like my child recently started of this medicine, he’s behaving this way, does anybody has any ideas. Sometimes they ask about doctors, I am living at this state, do you know any neurologist around there? This can be a localized context where you are asking also. I don’t think the doctors in the states are relevant to us.

Father of an 8-year-old child with intellectual disability discussing in web-based support groups

In addition, forums that are tailored toward Singapore residents such as Facebook groups often elicit worries about privacy and security owing to the large number of non-caregiving members being able to potentially access the information provided. Many participants also highlighted their difficulty in purchasing or selling items related to their caregiving, such as mobility devices. Although there are platforms that exist where caregivers can sell, give away, or purchase disability-related items, such as “Carousell,” these websites are not tailored toward caregivers and sell a multitude of products that can make searching for items daunting and difficult to navigate.

…my daughter, she likes to bite objects, so that time her teacher asked me to purchase the chewing tube, I also don’t know where to buy the things sometimes, very difficult to find.

Father of a 10-year-old special needs child

**Table 1:** Demographic details of the participants taking part in the pre–app development survey and interviews.

Characteristics		Predevelopment survey (n=103)	Predevelopment interviews (n=29)
Age (years), mean (SD)		49 (10.5)	51 (8.3)
**Gender, n (%)**			
	Male	24 (23.3)	6 (21)
	Female	79 (76.7)	23 (79)
**Ethnicity, n (%)**			
	Chinese	78 (75.7)	22 (76)
	Malay	9 (8.7)	1 (4)
	Indian	6 (5.8)	2 (7)
	Other	10 (9.7)	4 (14)
**Care recipient disability, n (%)**			
	Autism	44 (42.7)	10 (35)
	Physical disability	28 (28.2)	8 (28)
	Intellectual disability (including dementia)	16 (15.5)	5 (17)
	Other	15 (14.6)	6 (21)
**Care recipient age (years), n (%)^a^**			
	Child (<18 years)	52 (50.5)	15 (52)
	Adult (18-69 years)	20 (19.4)	3 (10)
	Older adult (70+ years)	24 (23.3)	11 (38)

^a^Seven values are missing among those who took the survey in this category.

### App Development

**Figure 1:**
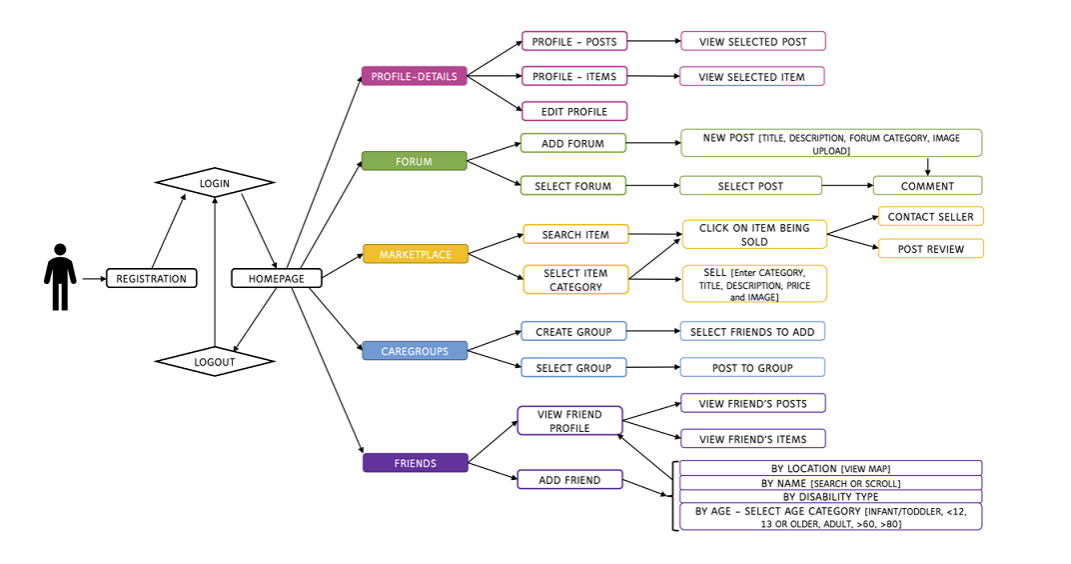
Flowchart of the Caregivers’ Circle app functions.

**Figure 2:**
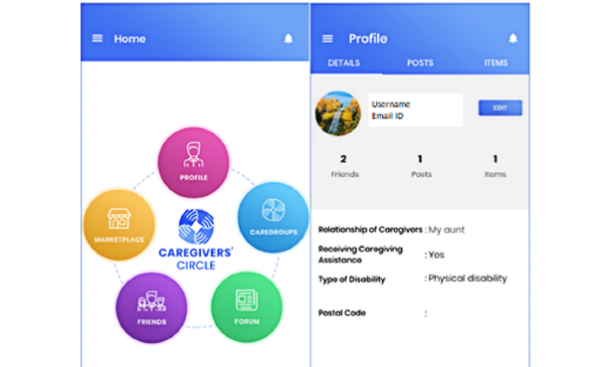
Screenshots depicting Caregivers’ Circle home page and profile page.

From the feedback garnered from the caregivers, the developers were able to identify several caregiver’s needs and gaps within the current support networks. This feedback was integrated into the mobile app called “Caregivers’ Circle” upon development. Features include *caregroups* to hold private discussions with friends or other users in the app, a *marketplace* to buy and sell items, a *friends* feature to search and add new caregiving friends, and a public *forum* for community discussions. These features are shown in Figure 1. The majority of the Singaporeans are Android users (59.7%), with iPhone operating system (iOS) users being the second most popular mobile platform (39.2%) [25]. Therefore, the community network app was developed for use on both Android and iOS platforms to provide broad accessibility across Singapore. Participants who wish to take part in the app functions will be able to download it from either Google Play or iTunes app stores. Within the Android platform, the integrated development environment Android Studio 3.4.1 was used to develop the mobile app using the programming language Core Java. Within the iOS system, the integrated development environment Xcode 10.1 was used to develop the app with the programming language Swift 4.2 and the package manager Cocoa pods. This is a development environment designed by Apple for iOS systems. Cocoa pods assist in the management of libraries organizing the source code that the Xcode tool uses. The backend of Caregivers’ Circle used the free script language Personal Home Page: Hypertext Processor 7.2 and Laravel 5.5, which is a web app framework. Upon downloading the app, users will be asked to register with their contact details, who they are caring for, whether they receive assistance in caring for their care recipient, the type of disability they are caring for, and the age of the care recipient. Privacy was a major worry among caregivers within Singapore and therefore, user contact details will be kept hidden and a pseudonym username can be used if they wish to remain anonymous. Profiles can be edited if the user wishes to change any detail (Figure 2). Within their profiles, users can select tabs to view any forum posts they have created or any items they have or wish to sell in the marketplace.

### Forum Feature

Within the predevelopment research, one of the prominent needs identified was that caregivers want to be able to discuss caregiving within a community of similar minded people within Singapore. Most existing platforms consist of caregivers from other countries who do not discuss locally relevant issues such as government schemes or provisions. Caregivers’ Circle will only be accessible to Singapore-based users to ensure that the app is locally relevant. Users can post on public forums categorized by disability type such as autism, physical disability, intellectual disability, hearing impairment, visual impairment, and multiple disabilities. The forums are open to any caregiver within Singapore registered in the app and can be used to discuss any issue or ask any question they think others in the community could assist with. Users can post links to articles, post photos, and write new posts. They can also comment on other users posts and create new forum categories for more specific discussion themes.

### Marketplace Feature

**Figure 3:**
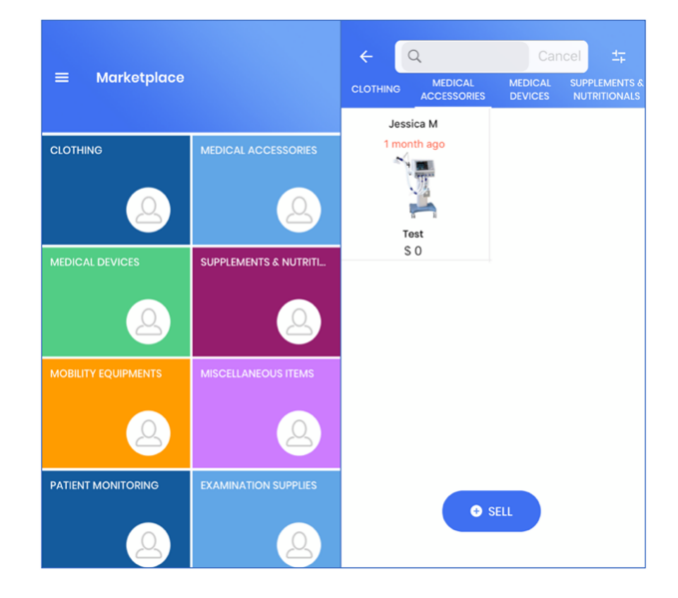
Screenshots depicting marketplace categories and item listings view.

Many caregivers lamented that they had difficulty finding or giving away items related to their care recipient, which led to the marketplace feature being developed (Figure 3). Within the marketplace, users can sell, give away, or buy items from other registered users. The items are categorized by different characteristics such as clothing, medical accessories, and mobility equipment. If a user is interested in purchasing an item, they can chat with the seller privately within the app to discuss the details of the purchase, pick-up location, etc. Sendbird Chat was used to create the chat function for users to talk to sellers in Marketplace. SendBird is a real-time chat and a messaging software development kit that has an easy-to-use and customizable user interface. 

### Caregroups Feature

**Figure 4:**
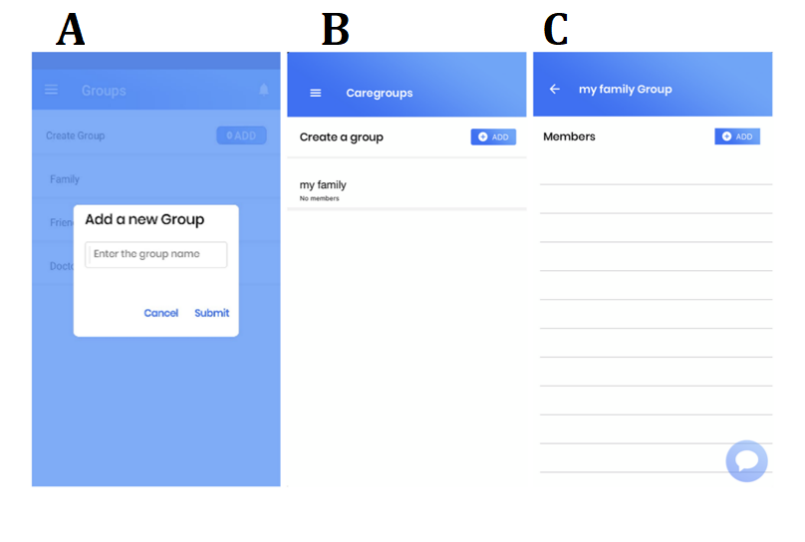
Screenshots depicting the functions of A. creating care groups, B. viewing list of care groups, and C. adding members to care groups and starting group chats.

Users can also post in private groups that are self-made called as caregroups (Figure 4). This feature allows users to talk privately to any other users within the app. Joint caregivers of a care recipient such as family members can use this feature to discuss specific needs related to their care such as organizing hospital visits and posting information regarding the care of their recipient and any private matters they wish to discuss. If users become friends within the forums and wish to talk privately, they can create a private group. There are no limits to the number of friends that can be added to a group nor the number of groups that can be created.

### Friends Feature

**Figure 5:**
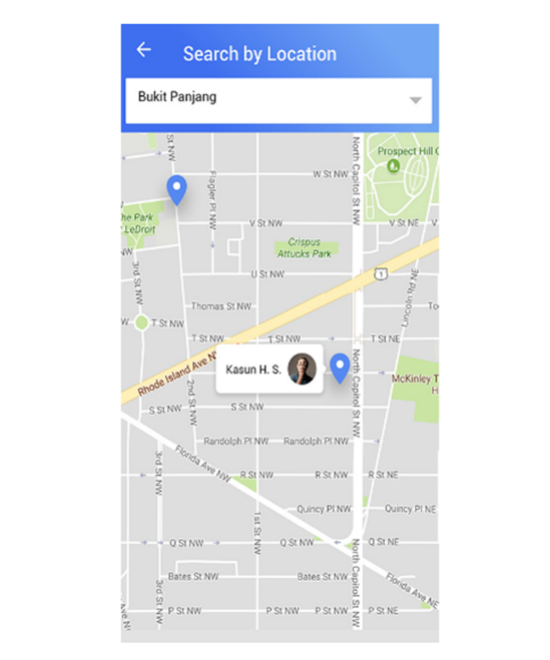
Screenshot depicting "Search by location" function for users to find other caregivers to befriend.

Loneliness is a major issue among caregivers who do not know where to go to find like-minded individuals going through similar life experiences. Within the app, users can search for new friends based on several key indicators. If the user knows the username of another caregiver, they can search for them by their username. If the user wishes to become friends with someone who is located near to them to make it easier to meet up in person or to discuss locally situated services, they can search for other caregivers via a location map (Figure 5). To create the mapping feature to search for friends, the Google Maps Software Development Kit was used along with Google Places. Both these platforms allow the app to provide geographic-based location services if the user wishes to divulge such information. Users can also search for friends by the care recipients’ disability type and the care recipients’ age, which are both entered by each user upon registration. This filter can allow caregivers to approach and befriend caregivers with similar needs. Once they become friends, users can view their friends’ details such as who they are taking care of, whether they are receiving assistance, how many friends they have, the number of forum posts they have created, and the number of items posted within the marketplace. Users can also click on the created posts of their friends or items they are selling. To ensure that the app is running smoothly, Crashlytics was used in both the Android and iOS systems. The Crashlytics crash reporter is a real-time software development kit that tracks, prioritizes, and fixes any stability issues that affect the app quality. The participants who took part in the pre–app development survey were asked to evaluate the app prototype. In addition, the caregiving organizations that were previously contacted for recruitment were contacted again to further enlist participants. Owing to time constraints during development, some participants could not download the app into their phones but were asked to either view screenshots of the app, which were then explained further by interviewers, or were guided through the app on the interviewers’ phone. However, some participants were able to test the app for 6-8 weeks. Participants were then asked their opinions on the app—whether they would use it and what could further be improved. The interviews were audio recorded with consent from the interviewee and were later transcribed for analysis.

### Usability Testing Results of the App

The prototype underwent the initial assessment by 32 end users. The users included caregivers of children with special needs and older adults with mental or physical disability (Table 2). The usability testing was conducted after seeking approval from the institutional review board (IRB-2018-11-016).

**Table 2:** Demographics of the app testers (n=32).

Characteristics		Values
Age (years), mean (SD)		43.2 (11)
**Gender, n (%)**		
	Male	10 (31)
	Female	22 (69)
**Ethnicity, n (%)**		
	Chinese	24 (75)
	Malay	2 (6)
	Indian	5 (16)
	Other	1 (3)
**Care recipient disability, n (%)**		
	Autism	9 (28)
	Physical disability	7 (22)
	Intellectual disability (including dementia)	9 (28)
	Other	7 (22)
**Care recipient age (years), n (%)**		
	Child (<18 years)	13 (41)
	Adult (18-69 years)	13 (41)
	Older adult (70+ years)	6 (19)

### Overall Look and Feel of the App

Overall, most participants stated that they would definitely download and try the app if it was available. They also mentioned that the look and feel of the app was neat and easy to navigate. Most users also said that the app will be useful to them.

…For me, I usually am the one who likes to have everything in one platform, I haven’t really found that.

Mother of an 11-year-old special needs child, speaking about the overall features of the app and previous apps in the market

…Oh, I would, I would definitely use that, yeah, because there is so much more information which I may need in the future which I don’t even know what the question is going to be. So, when the time comes, yeah, it will just be very useful.

Daughter taking care of an 89-year-old bedridden mother

### Usability of Forum Feature

Participants mentioned that the way in which the forums were designed was convenient and valuable. Care recipients may have a varied set of problems, many of which may require unorthodox solutions. They felt that the forum could connect them with other caregivers within Singapore who may be going through similar experiences and are able to share and learn from one another. Users also mentioned that forums could also help them in providing local information that international groups cannot provide.

…This is definitely useful; this is actually a part of sharing I think it’s most useful. Because very often you don’t go to doctor for something minor, in fact we never go to doctor for things like how do you address the fact that your kid keep grooming, how do we control that. Sometimes like questions like this you get tips from people who has been successful in trying out something, whether something they have read or something they accidentally bumped into which can be a solution.

Mother of a 16-year-old special needs child

…Yeah I think that will be very useful…Because, I previously went with my mom to a weekly session run by NNI … and then I met some of the daughters and sons of the patients and then we were chatting, and, you know, we find that we have common problems of course, yeah, but after that thing ended we just didn’t (we didn’t catch up), there was no platform to keep in touch, I guess.

Daughter taking care of an 80-year-old mother with dementia

### Usability of Marketplace Feature

Many participants agreed that the marketplace was a good feature in the app as these participants frequently found it difficult to find specific products for their care recipients. They envisioned that it could also be a place where they could give away/sell preloved items that they no longer need. Many participants highlighted the fact that the marketplace feature caters toward caregiving and care-recipient needs, unlike other general selling sites; this was received positively as the marketplace helped them to find specific products.

…the market is also quite good because sometimes this people, their parents passed away already, they got wheelchair they got bed, then they can come in here and sell or even free. You know? So, they can post and then easier sometimes, you ask people, nobody wants also.

Daughter of an 86-year-old mother with dementia

…I also like the marketplace, because we don’t know where to buy the things sometimes, very difficult to find.

Mother of a 10-year-old special needs child

…I like the concept of market place because overtime you do have stuffs that perhaps you don’t use anymore or you buy extra off, then you don’t need them anymore and it’s a waste. You can follow the Marie kondo method if you don’t want to throw it away, so this will be something great, and it helps other, even if it’s a giveaway, I think this will be a very nice thing.

Father of 15-year-old girl with intellectual disability

### Usability of Caregroups and Friends Features

Several participants stated that they do not have a lot of time to socialize and if there was a web-based community to share their experiences, it would be an easier way for them to make new friends at their own convenience. Some of the participants liked the “search by location” feature as they felt they could meet caregivers who are based geographically near to them and not lose time travelling to socialize.

…You get to know other parents with same condition kids, very difficult to make a new friends also.

Mother of a 10-year-old special needs child

…I’m very impressed with the map, and like for me who was needing, I think I’m surely going for the one. And this one was also quite good, because for us, we have other need, a special need, I think this would be very useful for me who don’t have much time.

Mother of a 5-year-old special needs child

…one good thing is if there’s critical mass of people in this, and then we can search by location by disability, it’s easier to reach out to the same people, people in the same situation and that’s something that we don’t really have now.

Mother of an 11-year-old child with autism discussing about the finding friend’s location-based searching

Users also highlighted few features that could be improved and added to the app to enhance the app use. Even though some participants liked the “search by location” feature in the app, others felt that this feature may be a concern for privacy. Although location was an optional feature based on initial interviews with caregivers, a few participants still raised concerns regarding general privacy in the app as they may be sharing information about vulnerable groups and suggested that if the app is supported and hosted by reputed organizations, it would help them to gain more trust.

…really, I would say that the tools are there… I think that’s really helpful because myself and quite a lot of people will be feeling quite reassured (organization name) is well-known and is getting an interest in it (app), may be more willing to more actively participate in such event or activity because there is some sort of body that’s supporting. It could be a communication portal for them to reach out to customers like us with their services. And whatever information that comes from there…It comes from an official body, or partners who is their official body who can share I’ve got this workshop or promotion for special trust one.

Father of a 15-year-old special needs child

Although participants were pleased with the community aspect of the app in assisting to alleviate some mental stress, some participants suggested including features such as inspirational quotes or messages and guides to motivate caregivers to remind them to take care of themselves and suggest simple ways on how they could destress and relax in their busy lives to enhance the current app.

…Ya, self-care. Self-care, I need a lot of that. I really want to make it, but I don’t know who. I don’t know how.

Mother of a 5-year-old special needs child

…You know caregivers are very stress, so I hope that’s something like music to distress me, would there be something to like there to, something like maybe fun. Maybe games, music, or something else. You know when you want to burst right, you need something to cool down.

Mother of a special needs 5-year-old child

Currently, the app is intended to accommodate all caregivers, although there are a few features such as the forums that can be tailored toward specific caregiving groups. Few users mentioned that it would be good if the app was further customizable according to their care recipients’ needs.

## Discussion

Caregivers face many burdens in life and often struggle to cope, feeling like they lack support or people to talk to. With time constraints and convenience issues, many caregivers cannot participate in face-to-face events that are often run by nonprofit organizations or the government and find that web-based groups tend to cater toward global or Western audiences rather than locally based Singapore residents. The rise of technology and, in particular, mobile phones has created the opportunity to utilize technology to serve the caregiving community. The development of the Caregivers’ Circle app incorporated caregiver feedback and previous literature to create a unique and usable community network app.

Overall, users were positive about the app and were confident that it could help them to live a better quality of life daily. The forums were seen as a positive space to discuss caregiving issues, make new friends, and answer caregiving queries to make them feel less alone in their situation. The marketplace was also well received as a place for caregivers to buy and sell or give away items related to caregiving that may be difficult or expensive to find elsewhere. There were mixed reviews about friend searching by location, with some caregivers seeing it as a way to make local friends and meet new people without having to travel far, while some caregivers were wary about the privacy issues that can arise from knowing a person’s geographical location. Users also liked the caregroups feature, which is similar in its function to other messaging apps, but with the added bonus of being contained within 1 caregiving platform to prevent the need to have several different apps. They also provided valuable feedback to improve the app to enhance usability. Taking user feedbacks into consideration on improving the features in the app, first, the research team aims to collaborate with trustworthy organizations that can help to launch the app to build trust with the users and to protect their privacy. Second, suggested features such as self-care tips, relaxing music, and inspirational messages to motivate the users will also be incorporated in the app. One of the limitations of this study was that owing to the delays in the development, the usability testing of the app for many participants could only be done using either screenshots or testing it for a short while on the interviewers’ phone. Future testing should investigate the usability of the app over a longer period of time to truly gauge the longevity and sustainability of the app. Upon taking the user feedback into consideration, further testing should also investigate the effects of the app on caregivers’ quality of life and the effect on loneliness over a longer period of time to evaluate the impact of the app.

In conclusion, our paper discusses the development of Caregivers’ Circle, an app that integrates ideas from caregivers, previous literature, and that uses new technological solutions to create a novel app for easier caregiving. Caregivers’ Circle is unique in its integrated approach. By integrating many features that caregivers need on a daily basis into an easy app can save time as well as provide help to navigate their caregiving smoothly.

## Abbreviations

iOS: iPhone operating system
